# The quick pivot: Capturing real world modifications for the re-implementation of an early psychosis program transitioning to virtual delivery

**DOI:** 10.3389/frhs.2022.995392

**Published:** 2023-02-03

**Authors:** Wanda Tempelaar, Nicole Kozloff, Allison Crawford, Aristotle Voineskos, Don Addington, Tallan Alexander, Crystal Baluyut, Sarah Bromley, Sandy Brooks, Lauren de Freitas, Seharish Jindani, Anne Kirvan, Andrea Morizio, Alexia Polillo, Rachel Roby, Alexandra Sosnowski, Victoria Villanueva, Janet Durbin, Melanie Barwick

**Affiliations:** ^1^ Slaight Family Centre for Youth in Transition, Centre for Addiction and Mental Health, Toronto, ON, Canada; ^2^ Department of Psychiatry, Faculty of Medicine, University of Toronto, Toronto, ON, Canada; ^3^ Institute of Health Policy, Management and Evaluation, Dalla Lana School of Public Health, University of Toronto, Toronto, ON, Canada; ^4^ Virtual Mental Health and Outreach, Centre for Addiction and Mental Health, Toronto, ON, Canada; ^5^ Campbell Family Mental Health Institute, Centre for Addiction and Mental Health, Toronto, ON, Canada; ^6^ Department of Psychiatry, University of Calgary, Calgary, AB, Canada; ^7^ Centre for Addiction and Mental Health, Toronto, ON, Canada; ^8^ Provincial System Support Program, Centre for Addiction and Mental Health, Toronto, ON, Canada; ^9^ Child Health Evaluative Sciences, SickKids Research Institute, The Hospital for Sick Children, Toronto, ON, Canada

**Keywords:** implementation, adaptations, virtual mental health, early psychosis, modifications, youth

## Abstract

**Background:**

Team-based Early Psychosis Intervention (EPI) services is standard of care for youth with psychosis. The COVID-19 pandemic required most EPI services to mount an unplanned, rapid pivot to virtual delivery*,* with limited guidance on how to deliver virtual clinical services or whether quality of re-implementation and treatment outcomes would be impacted. We used a structured approach to identify essential modifications for the delivery of core components and explored facilitators and barriers for re-implementation and fidelity of a virtually delivered EPI intervention.

**Materials and methods:**

NAVIGATE is a structured approach to team-based EPI. It provides detailed modules to guide delivery of core components including medication management, psychoeducation and psychotherapies, supported employment/education, and family education. Having initially implemented NAVIGATE at the Centre for Addiction and Mental Health (CAMH) in 2017, the EPI service transitioned to virtual delivery amid the COVID pandemic. Using a practice profile developed to support implementation, we detailed how core components of NAVIGATE were rapidly modified for virtual delivery as reported in structured group meetings with clinicians. The Framework for Reporting Adaptations and Modifications for Evidence-Based Interventions (FRAME) was used to describe modifications. Fidelity to the EPI standards of care was assessed by the First Episode Psychosis Fidelity Scale (FEPS-FS). Re-implementation barriers and facilitators and subsequent mitigation strategies were explored using structured clinician interviews guided by the Consolidated Framework for Implementation Research (CFIR).

**Results:**

Identified modifications related to the intervention process, context, and training. We identified contextual factors affecting the re-implementation of virtually delivered NAVIGATE and then documented mitigating strategies that addressed these barriers. Findings can inform the implementation of virtual EPI services elsewhere, including guidance on processes, training and technology, and approaches to providing care virtually.

**Discussion:**

This study identified modifications, impacts and mitigations to barriers emerging from rapid, unplanned virtual delivery of EPI services. These findings can support delivery of high-quality virtual services to youth with psychosis when virtual care is indicated.

## 1. Introduction

Early psychosis intervention (EPI) is an evidence-based treatment that has become the standard of care for youth with psychosis (1). EPI care is provided by a multidisciplinary team who provide comprehensive treatment including psychoeducation and psychotherapy for psychosis (most commonly, cognitive behavioural therapy), case management, individual psychopharmacological intervention, family education and support, and support for education and employment (1, 2). Previous EPI effectiveness studies demonstrated superior outcomes including reduced mortality, decreased risk of relapse, fewer hospital readmissions, and increased employment rates relative to care as usual (3–7). Furthermore, evidence shows that a manualized package of EPI services called NAVIGATE results in improved functional outcomes compared to care as usual. Clients receiving NAVIGATE showed greater improvement in quality of life and psychopathology, greater involvement in work and school, and remained in treatment longer compared to clients receiving community care (2).

EPI models of care, such as NAVIGATE, are designed for in-person delivery, emphasizing frequent contacts and community outreach. The COVID-19 pandemic prompted an abrupt shift to virtual delivery of EPI care to ensure continuity in the face of public health restrictions (8–10). However, little was known about the modifications required to provide EPI care virtually or their impacts. The abrupt need for virtual care delivery without suspending service meant there was no time for planning or training to prepare for this shift. Clinicians and clients had to quickly adapt to a new delivery method with ongoing adjustments occurring over time.

The impacts of these modifications and whether virtual delivery of EPI care would achieve the same benefits as the in-person intervention were unknown. With the shift to virtual delivery, it is important to better understand the nature of the modifications that are made and their impact on treatment delivery and outcomes. Modifications, especially if unplanned, may or may not align with the core components required to ensure the intervention is effective (11). For instance, modifications that alter or remove core components of the EPI model, or fail to align with population needs may reduce the effectiveness of virtual EPI compared to the original, in-person intervention (11, 12).

Previous work on investigating modifications of evidence-based interventions led to the development of frameworks that can be used to systematically describe and evaluate modifications to evidence-based interventions, including the Framework for Modification and Adaptations (11, 13). The FRAME captures characteristics of modifications and was recently updated to include broader aspects of the implementation process, such as reasons for the modifications (e.g., to improve feasibility, engagement, outcome), level of the modifications (client, clinician, program), timing of the modifications (prior, during, for scale up), and fidelity to the original intervention (consistent or inconsistent) (11). This detailed framework facilitates understanding of the relationships between the modification and key outcomes that can be tested in implementation studies (14, 15). This is important because modifications that remove or alter core components of an intervention may be less effective. Despite significant developments to identify and classify modifications and their impact on outcomes using structured frameworks, there is little guidance on how to systematically document (ad hoc) modifications in a dynamic setting, how to assess the impacts of these modifications over time, and how contextual factors relate to modifications and outcomes.

The Centre for Addiction and Mental Health (CAMH) in Toronto, Ontario, is home to the largest EPI program in Canada, providing assessment and ongoing services to people aged 14–29 years who present with early psychosis. CAMH implemented the NAVIGATE model for EPI service delivery in 2017 for all clients attending the EPI outpatient clinic, and is currently leading a multisite implementation effectiveness study of NAVIGATE across EPI programs in the province of Ontario (16). NAVIGATE is expected to increase consistency of delivery and improve program fidelity to EPI practice standards (16). CAMH has a dedicated Virtual Mental Health and Outreach program that provides telepsychiatry to clients in remote and rural areas. During the COVID-19 pandemic, this program expanded to support other CAMH programs in their delivery of virtual care.

Soon after the onset of the pandemic, CAMH's Slaight Centre Early Intervention Service (SCEIS) was awarded COVID-19-related research funding to investigate the re-implementation of NAVIGATE from in-person to virtual delivery. The aims of this study are (1) to identify the modifications required to re-implement and deliver the NAVIGATE model virtually, (2) to assess whether these modifications affected fidelity to the EPI practice standards, (3) to explore implementation facilitators and barriers related to *re-implementation*, a term coined here to reflect a second implementation effort following the earlier, full implementation of an intervention, (4) to examine satisfaction with virtual delivery of NAVIGATE among clients, family members and clinicians, and (5) to investigate service engagement with virtual delivery of NAVIGATE. To address these aims, we conducted a mixed methods study using a convergent study design to investigate the unplanned shift to virtual delivery of EPI (17). The current manuscript addresses aims 1, 2 and 3, and illustrates the application and utility of a practice profile (18), the FRAME framework for identifying and documenting model adaptations and unanticipated impacts (11, 13), and the Consolidated Framework for Implementation Research (CFIR) for identifying barriers to re-implementation (19). Objectives 4 and 5 related to outcomes will be reported separately.

## 2. Materials and methods

### 2.1. Design

The study used a mixed methods, pragmatic, implementation and evaluation design described in more detail elsewhere (20). Youth and family members with lived experience, front-line clinicians, and clinical administrators were engaged in a structured, stepwise approach to track adaptations needed to provide NAVIGATE care virtually. Structured approaches were used to evaluate re-implementation outcomes as measured by fidelity, and to explore implementation facilitators and barriers. Throughout this manuscript we refer to “virtual” delivery of care when care is provided via phone or tele/videoconference.

## 3. Study setting and population

This study was conducted at SCEIS, the outpatient EPI program at the Centre for Addiction and Mental Health (CAMH) in Toronto, Canada. SCEIS serves people aged 14–29 years old who present with early psychosis (schizophrenia, schizoaffective disorder, schizophreniform disorder, bipolar I disorder or major depressive disorder with psychotic features, substance-induced psychotic disorder, unspecified psychotic disorder). Located in downtown Toronto, Canada, this EPI service is staffed by approximately 40 clinicians who assess approximately 600 new clients annually.

The Ontario Ministry of Health provides coverage for all medically necessary services including EPI to residents through the Ontario Health Insurance Plan (OHIP), and this coverage was maintained in the transition to virtual care.

SCEIS provides EPI services according to the NAVIGATE model, a highly structured program of coordinated specialty care with clearly defined roles for staff (21). Initially implemented at CAMH in late 2017, the model consists of four core clinical roles: Individual Resiliency Training (IRT), Supported Employment and Education (SEE), Family Education Program (FEP), and individualized medication management (21). Additional core components that are fundamental to the NAVIGATE program include: Team Lead who facilitates monitoring; Practice Feedback and Training; and Caseloads small enough to allow for the intensity and frequency of required contact. Manualized protocols are used to operationalize current EPI standards, and all clients are systematically offered all treatment components with regular team meetings to review client progress, fidelity, and need for adjustments. All clients receive substance use support as part of the IRT manual. Where there is additional need for substance use support beyond the general manual, clients can receive specialized support from a clinical psychologist at SCEIS or from additional programs at the substance use disorder services at CAMH.

### 3.1. Stakeholders

This study engaged youth and family members with lived experience, front-line clinicians and administrators according to current best practices (22). Stakeholders contributed meaningfully to the study design, data collection, integration of findings and knowledge dissemination. We held monthly meetings with the principal investigators, operational research staff, youth and family members with lived experience, front-line clinicians and clinical leads (“steering committee”) to review the progress of re-implementation and data collection, and to plan for knowledge dissemination. Monthly “knowledge user meetings” were held during the first phase of the study with front-line clinicians and clinical leads to discuss program modifications and their impacts, barriers to virtual care delivery, clinician resources and training.

### 3.2. Context: the COVID-19 pandemic

The shift to virtual delivery of care occurred abruptly in March 2020 due to COVID-19-related public health directives to stay at home during the first COVID-19 lockdown in Toronto. The first COVID-19 lockdown lasted from March to June 2020 (with ongoing restrictions persisting to varying degrees until the time of submission) and prompted a hospital-wide transition to virtual delivery for most outpatient services. Exceptions were made to allow in-person appointments for a small number of clients for whom virtual assessment and treatment was not feasible (e.g., clients in crisis and/or requiring a hospital admission, those receiving intramuscular injections, and/or those lacking access to virtual care).

The abrupt shift in the modality of care delivery pre-empted any preparation and planning for this transition. Fortuitously, several facilitating events occurred. Prior to March 2020, CAMH had taken steps towards integrating a digital platform to enhance capability for virtual meetings and enable the use of virtual care throughout the organization. After an extensive process, a digital platform (Cisco Webex) was chosen that met the Ministry of Health's privacy and confidentiality requirements including safeguarding Personal Health Information of clients. Proof-of-concepts in clinical and non-clinical settings had been conducted with this digital platform prior to the COVID-19 pandemic (23). Other enabling factors at CAMH that predated the pandemic included exclusive use of electronic medical records, and the transition to using laptops instead of desktop computers in order to facilitate remote and mobile work.

Once the pandemic triggered the shift to virtual care, CAMH rapidly scaled the deployment of the Cisco communications platform and initiated organization-wide training for clinicians in the use of Cisco Webex and the Ontario Telemedicine Network (OTN), two provincially approved digital platforms for providing virtual care. This training was provided to over 400 CAMH clinicians.

CAMH developed and implemented a virtual care policy and protocol that covered procedures for providing care in a virtual setting such as privacy, confidentiality, documentation practices, and practical instructions for providing virtual care. Subsequently, the Virtual Mental Health and Outreach team developed digital mental health training for clinicians on delivering virtual care in clinical settings. Training content included the context and evidence base for virtual care; clinical experiences; individual and group settings; safety and confidentiality procedures; technology; and the therapeutic relationship in a virtual setting (24). Other tools for facilitating virtual care delivery were made available across CAMH including a communications application allowing for instant messaging and phone calls with other team members and clients (Cisco Jabber); a secure file transfer platform to share files; and applications for faxing and scanning documents remotely. CAMH EPI clinicians were provided with mobile phones to facilitate voice communications and text reminders with clients.

Virtual care was enabled across Ontario by a shift in the Ontario Ministry of Health billing codes and requirements to enable remuneration of virtual care (via videoconference or phone) provided by physicians.

## 4. Procedures

### 4.1. Objective 1: modifications

Our approach to documenting modifications included the use of the NAVIGATE practice profile (18, 25) and the FRAME framework (11). A practice profile is a tool for describing the core components of an innovation or model of care, including the principles that underlie the model. Core components are prescribed by the innovation developer but how each core component is executed and by whom is determined by the implementing organization to guide implementation and delivery. Core components are the features of a model or intervention that must be present to ensure that it is delivered as intended to achieve expected outcomes. The profile provides a structure for documenting variations to the innovation as well as implementation outcomes. Once an innovation is described in sufficient detail, effective implementation methods can be applied to explore the organizational functions needed, develop staff competencies, monitor data for continuous improvement and sustainment, and ensure that leadership and administrative practices remain facilitative.

**Figure 1:**
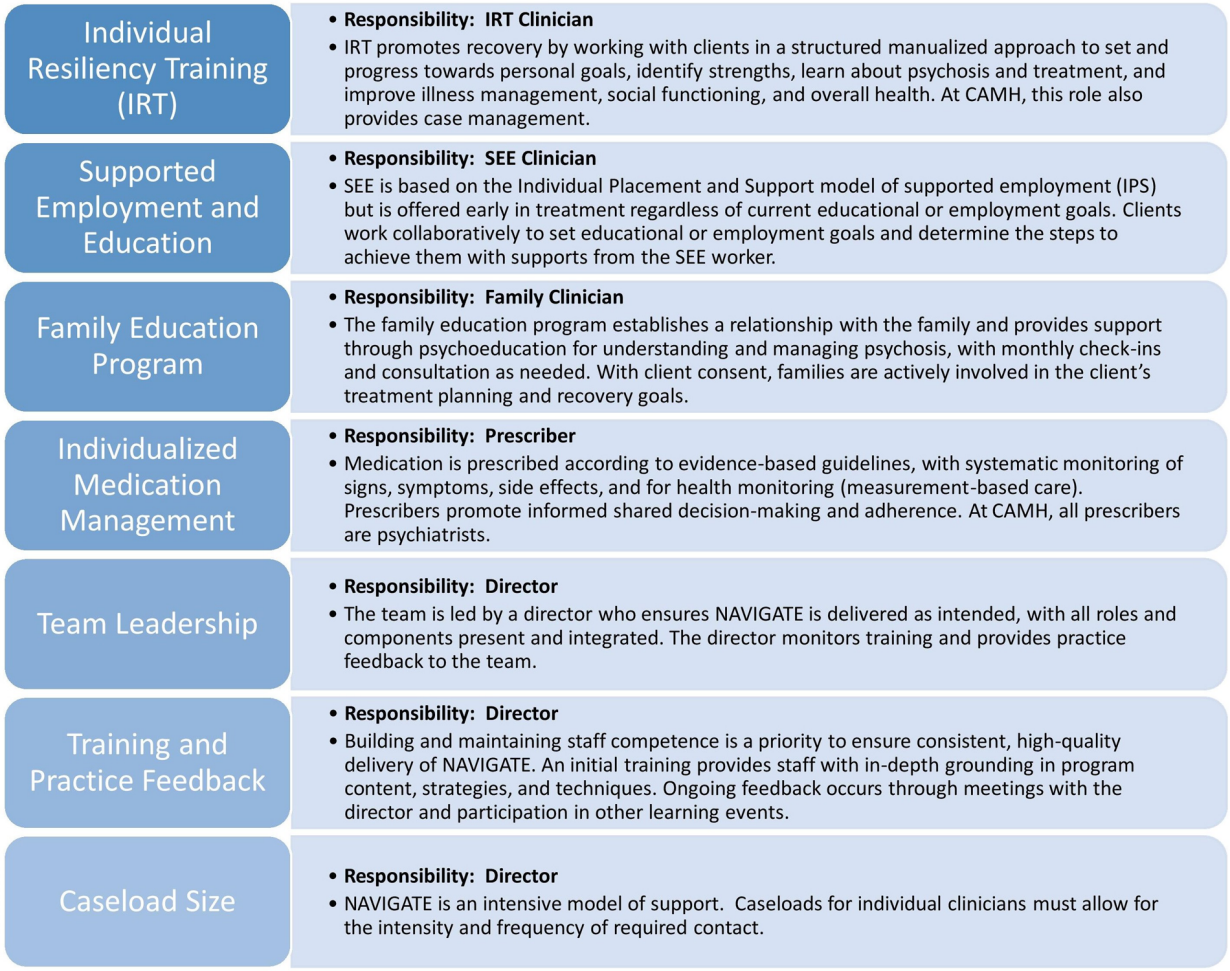
NAVIGATE core components.

Prior to the pandemic, research team members developed a NAVIGATE practice profile (26). This development took place through an iterative process that included a review of key NAVIGATE manuals and other model documents, published articles from the RAISE-ETP study that developed and first implemented NAVIGATE, as well as feedback from clinicians and implementation specialists familiar with the model (21). A penultimate draft was reviewed by model originators, further revised and finalized. The final practice profile identified seven core components: Individual Resiliency Training (IRT), Supported Employment and Education (SEE), Family Education Program (FEP), Individualized Medication Management, Team Leadership, Practice Feedback and Training, and Caseload (Figure 1). We used this NAVIGATE practice profile to describe and document modifications for each core component in the current study. We adjusted the descriptions of how the components were delivered virtually and added information on mitigation strategies that were taken to facilitate the change or to reduce potential negative impacts and amplify positive impacts of the modifications. Structured reflection sessions were conducted remotely with clinicians in each NAVIGATE role (IRT, SEE, FEP, prescribers, team lead) during the re-implementation process to document modifications and impacts. At each discussion, we monitored challenges, contextual factors, and impact and tracked subsequent modifications or mitigating strategies. From these discussions we were able to identify the reasons modifications were made and at what level they occurred. This method of tracking modifications in structured reflection sessions has previously shown potential as a straightforward and low-burden approach for documenting events across a dynamic implementation setting (27). Sessions with the clinicians and the clinical manager occurred at 2–3 and then again at 12 months into the study. Interim updates by clinician representatives were provided as part of monthly meetings throughout the first year of the study and clinicians representing different NAVIGATE roles reviewed and finalized the modifications described in the practice profile. Barriers identified during the initial group sessions were reviewed by the research team to inform new adaptations for enhancing the re-implementation process.

Modifications to the practice profile were then coded using the FRAME to document underlying process, rationale and purpose (11). For our context of re-implementation, we added an additional factor to capture the “effects” of modifications. We identified potential and realized positive, neutral and/or negative intended/unintended effects of modifications and described mitigating strategies that were undertaken to lessen negative impacts, if applicable. To document the “reasons” underlying each modification, we added the specifier “COVID-19 pandemic” as the “outer setting context” to indicate why the modification was made. Documenting modifications in response to culture was not applicable to our context, as modifications were not related to the implementation of the intervention in cultures different from where the intervention was first implemented.

### 4.2. Objective 2: fidelity

Implementation fidelity refers to the extent to which an intervention is delivered as intended by the program developers and in line with the program model (28). In the present study, we used the First Episode Psychosis Services Fidelity Scale (FEPS-FS) to assess fidelity to evidence-based practices for EPI delivery (29).

#### 4.2.1. Fidelity to EPI practices

The FEPS-FS is a validated measure of fidelity to the standards of EPI care (29). Scale development was based on a review of evidence combined with an expert consensus process and is not tied to any specific model of EPI delivery. Thirty-three items are rated on a 5-point scale from “not implemented” to “fully implemented.” A rating of 4 is considered satisfactory adherence. The scale is designed such that the items measure delivery in relation to the core components of the EPI model (adherence); quality of delivery using strategies such as clinician observation is not assessed (30).

The FEPS-FS items assess team structure (integrated approach), client continuity of care (early intervention, retention), and client receipt of medical and psychosocial treatments (comprehensive care). In Ontario, a community of practice for EPI programs, the Early Psychosis Intervention Ontario Network (EPION), developed a process to assess fidelity with this scale using a site visit methodology (26, 31). Fidelity ratings are based on interviews with staff, client chart review and administrative data and are usually made after a 1-to-2-day site visit by independent assessors. In this study, COVID-19 related restrictions required us to assess fidelity remotely via phone/video staff interviews and virtually trained on-site health record abstractors (32).

Fidelity assessments were done twice; retrospectively to capture practice prior to pandemic restrictions, when care was provided in-person (January–December 2019), and after the shift to virtual care delivery (July 2020–June 2021). For each assessment period, 10 client charts were randomly selected for clients enrolled in the program for at least one year during that period. These charts were abstracted by remotely trained on-site staff. Two independent fidelity assessors conducted phone/video interviews with program informants about NAVIGATE delivery during each of these periods. Both at the beginning and throughout each interview, the assessors reminded the participant about the practice period in question. For each period, interviews were held with the team lead, prescriber and 4 clinicians in different NAVIGATE roles. The assessors then reviewed the chart, interview and program administrative data to develop preliminary ratings that were discussed in a consensus meeting with a fidelity expert and then finalized.

Individual item ratings and the total mean score were reported for each period. Item ratings were grouped into one of five domains that pertain to: team structure, access and continuity, comprehensive assessment, medical treatments and psychosocial treatments.

### 4.3. Objective 3: implementation facilitators and barriers

Facilitators and barriers were captured with a CFIR informed semi-structured interview. The CFIR is a determinant framework of 39 factors known to influence implementation, categorized into five major domains: intervention characteristics; outer setting; inner setting; staff characteristics; and implementation process (30). Since CAMH clinicians had previously implemented NAVIGATE, the CFIR-informed interview focused specifically on the re-implementation of virtual delivery. We included 38 CFIR constructs, omitting cost as the delivery was part of standard care. We interviewed 8 clinicians (IRT, SEE, FEP, prescriber, team lead) by videoconference. Interviews were administered and coded deductively using a variation of the Rapid Analysis (RA) method, an alternative to in-depth analysis of interview data that allows for faster analysis and dissemination of implementation findings while using fewer resources (19, 33). Coding identified facilitators and barriers as well as the direction (valence) and strength of the association between factors and implementation success. For the first analytic step of the RA method, the analysts captured interview comments on a templated summary table in real time. The summary table aligned with the CFIR interview guide (domain and factors). The second analytic step involved assigning a valence rating to each factor to denote a positive or negative influence on implementation (+, neutral, −). Strength of the association was then rated (−2, −1, 0, mixed, +1, +2) and determined by a number of factors, including level of agreement among participants, strength of conviction, and use of concrete examples. In the last analytic step, memos were written to summarize the findings for each factor.

## 5. Results

### 5.1. Objective 1: modifications

#### 5.1.1. Cross-cutting modifications

Group meetings with clinicians revealed that three types of modifications needed for the virtual delivery of NAVIGATE were cross-cutting and independent of NAVIGATE core components, while others were unique to a core component. Cross-cutting modifications related to technology, procedures, and training.

Technological modifications included providing hardware and software to clinicians to facilitate remote work (including laptops and mobile phones), and the organization-wide roll-out of Cisco Webex, a digital platform for providing virtual care.

Procedural modifications related to privacy, safety and confidentiality guidelines for virtual care delivery which included obtaining client consent for virtual appointments, Mental Health Act certification procedures (i.e., for involuntary commitment), and changes to physician remuneration for virtual care.

Training modifications involved clinician orientation to new software applications including the digital platform used for virtual care, clinician training on building engagement with clients in the context of virtual care, provided by a youth with lived experience, risk assessment and addressing crisis management with clients in crisis, suggestions for providing trauma-informed care in a virtual setting, and considering health equity in virtual care delivery. Several of the cross-cutting modifications stemmed from decisions made at the organizational level and impacted the whole organization. For instance, changes made to the remuneration for provision of virtual care, a particularly relevant decision, was made at the provincial governmental level (Ministry of Health).

#### 5.1.2. Core component related modifications

We documented 26 modifications related to the four NAVIGATE core clinical roles: 8 modifications for IRT, 5 for SEE, 4 for FEP and 9 for the prescriber role (Tables 1a–1f). Most of these modifications occurred during the onset of the shift to virtual care delivery. About two-thirds of the modifications were unplanned or reactive modifications. Most modifications were made by clinicians and/or the clinical manager and occurred at the clinic/unit level (59%), although one-third occurred across the organization (31%) (Table 1a). Most modifications were unrelated to the content of the intervention (69%) and were consistent to the provincial standards for EPI care (63%). Overall, modifications served to increase or maintain client engagement (34%) and to increase and maintain client retention (28%) and improve feasibility of delivery (19%). Little changes were noted for the three NAVIGATE core components that were not directly related to a clinician role. The team leadership role continued as before the shift to virtual care, though all meetings were held virtually, including supervision and training. One of the functions for the team leadership role captured in the practice profile is community outreach, which includes providing targeted education to health, social service, or community groups. There were few community outreach activities, even before the COVID-19 pandemic, and this did not increase with the switch to virtual care delivery. Regarding training and practice feedback, no significant changes were noted to the onboarding process, other than a modality switch to virtual meetings and adding training on virtual delivery of care. The team meetings continued without changes in a virtual setting, and clinicians met virtually with the clinical lead or substitute weekly. With the switch to virtual care delivery there was an increased demand for training on how to use the virtual applications. Caseload size did not change, though workload increased, and mitigating strategies for the increased workload were captured in the Practice Profile.

**Table 1a:** FRAME Virtual NAVIGATE - Summary of 26 modifications (11). Adapted from: Marshall et al. 2021 (34). All items with an asterisk (*) were added by the current authors.

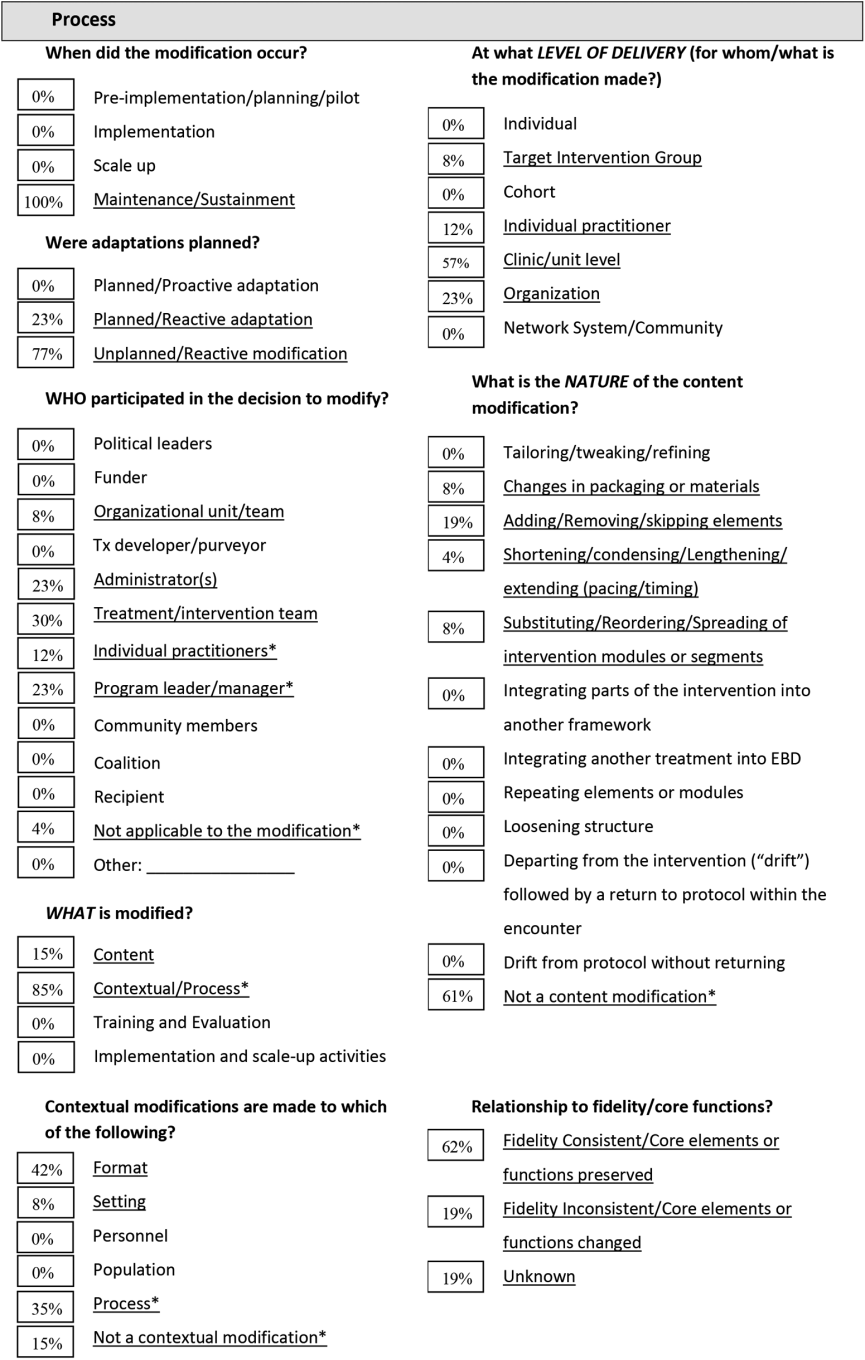
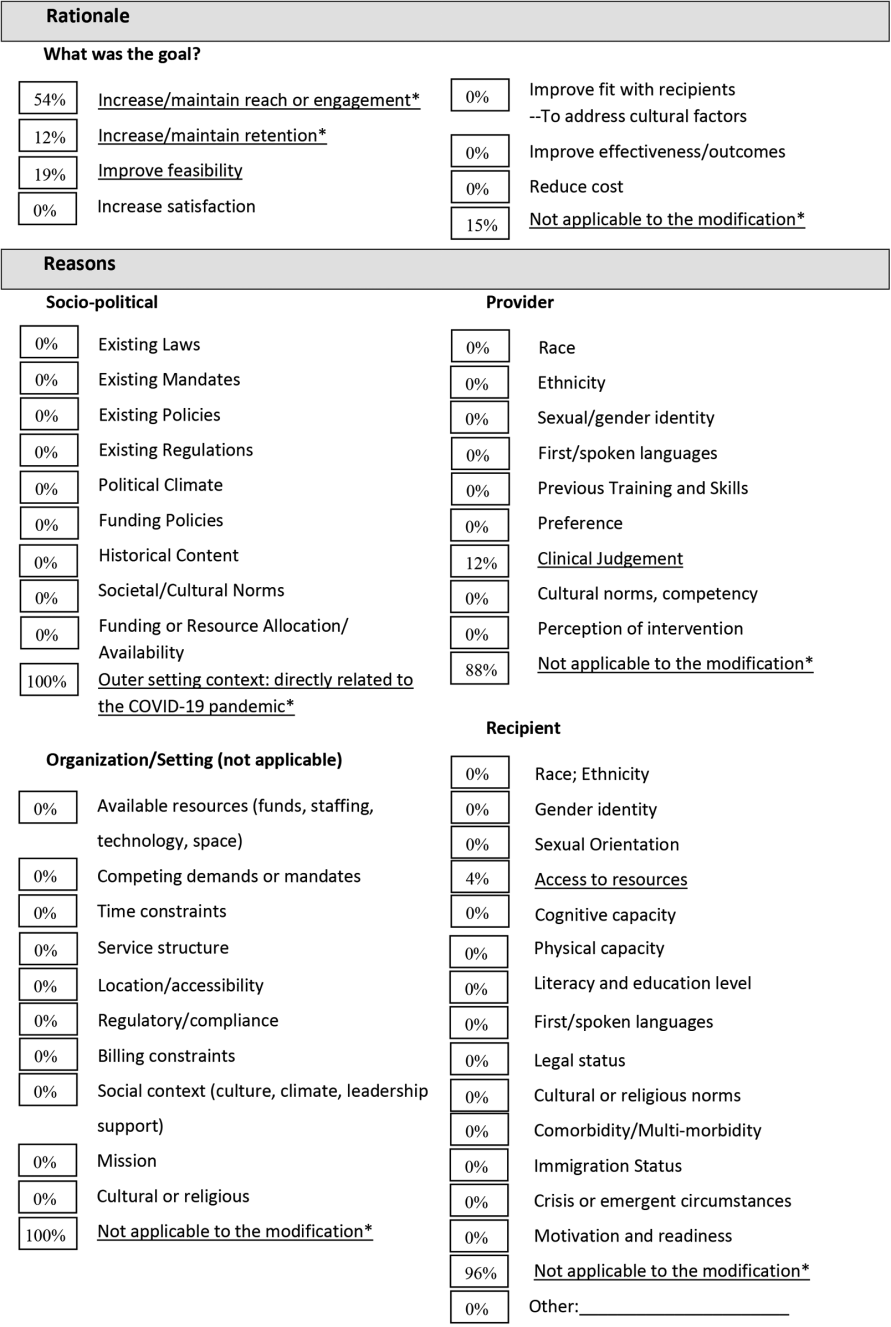

% shows the number of modifications per category as coded per the FRAME, divided by the total number of modifications.

#### 5.1.3. Modifications for individual resiliency training

Modifications to individual resiliency training (IRT) components occurred early during the shift to virtual care delivery and were largely unplanned and reactive to the shift to virtual delivery (Table 1b). Most decisions about modifications were made by the treatment team and the clinical manager, were fidelity consistent, and served to maintain client engagement or retention. For example, clients were offered shorter but more frequent appointments, appointment reminders were sent more often, and hardcopy worksheets and handouts from the NAVIGATE modules were replaced with fillable PDF files that could be shared with clients on the video screen during appointments. Modifications were intended to maintain delivery of the IRT core components despite restrictions to in-person practice. One advantage mentioned by the IRT clinicians was that they were able to gain insights into client's living situations when they attended via videoconference from home. Disadvantages of providing IRT care via videoconference or phone were a less fulsome assessment of nonverbal cues, and client and clinician challenges with technology, connectivity, and engagement during appointments. An increased workload for IRT clinicians occurred, partly due to training demands, but also related to increased communication with clients and clinicians (e.g., via email).

**Table 1b:** Brief report of 8 Virtual NAVIGATE modifications pertaining to the Individual Resiliency Training (IRT) role based on FRAME elements (11). Adapted from: Marshall et al. 2021 (34).

FRAME Elements	Brief report from virtual NAVIGATE IRT modifications
**Process**	
**When did the modification occur?**	IRT modifications resulted from the transition to virtual care due to the COVID-19 pandemic in March/April 2020. These changes occurred during the maintenance/sustainment phase of NAVIGATE delivery in order to continue to deliver care to clients throughout the pandemic by updating the mode of delivery (i.e., via phone/videoconferencing, and eventually adjusting back to in-person appointments as the provincial mandates permitted).
**Were adaptations planned?**	Modifications were primarily unplanned and reactive, resulting from the sudden onset of the pandemic. For instance, clients were offered extra appointments that were shorter in duration as well as more appointment reminders via email or SMS text messaging if needed. Planned/reactive iterative adaptations involved updating NAVIGATE materials and modules into PDF fillable files to share synchronously virtually, and training sessions provided to clinicians.
**Who participated in the decision to modify?**	The SCEIS program leader/clinical manager made most of the decisions on a clinic/unit level. Many partners contributed to decisions and were involved in making modifications relevant to the IRT role including members of the “virtual-NAVIGATE study team” SCEIS staff such as individual practitioners. Certain decisions around virtual care provision were taken on a hospital-wide or provincial level, involving administrators, and CAMH management.
**Adaptations**	
**What was modified?**	The process of delivering NAVIGATE was modified so that the continuity of care could be maintained safely in a virtual context in response to provincial mandates. This included providing staff with work cell phones to text and/or call clients, sharing materials via screen sharing instead of face-to-face, and involving the family member in the IRT session to improve access and activation. Training was offered to SCEIS clinicians on a wide range of virtual care topics (e.g., the technical aspects of using the virtual platform as well as addressing building engagement and ensuring privacy in a virtual setting).
**At what level of delivery were modifications made?**	The majority of modifications were made within the clinic/unit level at SCEIS. Some modifications made for the target intervention group included modifying material so that it could be shared with clients across EPI sites electronically.
**What was the type or nature of context or content-level modifications?**	Format changes pertained to transitioning from in-person appointments to delivering care virtually, making adjustments to virtual appointments that warranted in-person care, and altering the amount and length of appointments (i.e., extra appointments that were shorter in duration). Contextual changes included alterations in setting which changed from in-person (at SCEIS) to clients’ homes. Process changes involved sending more appointment reminders via email and text, with the ability to attach documents to Webex invites. Content modifications centred on modifying materials (e.g., creating fillable PDF files) as well as creating web-based resources to support the virtual delivery of NAVIGATE.
**What was the relationship to fidelity?**	The majority of modifications were fidelity consistent, as efforts were made to critically consider and preserve the core elements of the IRT role while making the necessary adjustments to continue delivery of care.
**Rationale**	
**a. What was the goal?** **b. What were the reasons?**	a. Modifications to the IRT role in order to deliver NAVIGATE virtually aimed to increase/ maintain client engagement, retention, and satisfaction as well as to improve feasibility. b. Reasons for modifying NAVIGATE to be delivered virtually largely centred around the outer setting context, namely, the pandemic. There were no specific organizational/setting, provider, or recipient reasons for this transition.
**Outcome**	
**a. What were the positive outcomes?** **b. What were the negative outcomes?**	a. Continuity of care could be maintained via phone (including texting) for those who do not have access to devices and/or with connectivity issues; less perceived stigma for clients by not having to come on-site (which can increase attendance); greater insight into client's living situations; less formal appointments which can enhance engagement; more joint appointments/”warm handovers” with other care providers; reduced length of appointments increased attention compared to longer virtual appointments and facilitated brief check-ins of clients’ symptoms while improving time-management for clinicians; improved fit to a virtual context and for the target population at SCEIS; increased collaboration between clients and clinical providers. b. Less fulsome assessments of mental health status/nonverbal cues and safety (especially when connecting via phone); unable to support clients going to the emergency department for crisis services; challenges with building/maintaining the therapeutic relationship; COVID-exposure risks for staff and clients who needed to come on-site; less boundaries and appropriate behaviour when meeting virtually; issues with technology and connectivity (which could lead to less time to connect); privacy issues; client mistrust of technology; increased clinician workload and appointments; less time for IRT and more focus on case management tasks.

To mitigate challenges introduced by modifications to IRT, the research team gathered web-based resources (websites, brief videos, mobile phone apps) related to the content of the IRT NAVIGATE modules to enhance client engagement in the virtual IRT sessions. These resources were selected by IRT clinicians and youth with lived experience and shared among IRT clinicians. To mitigate technological challenges, IRT clinicians connected with clients via phone to guide them on how to use the digital platform or encouraged clients to seek digital support from family members. To lessen the burden of training demands on clinicians, the team disseminated weekly, bite-sized information by email with practical tips on technology and procedures related to virtual care delivery and clinician wellness.

#### 5.1.4. Modifications for support for education and employment

Modifications made to the Support for Education and Employment (SEE) component were all unplanned (Table 1c). Modification decisions were mostly taken by the treatment team with some input from the clinical manager and individual clinicians. Most modifications were consistent with fidelity, with the exception of a reduction in clinician visits to community-based education and employment settings. SEE modifications included changes to how SEE clinicians were introduced to clients during IRT sessions, fewer opportunities for community outreach visits due to COVID-19 related restrictions, countered by more opportunities to organize and attend virtual meetings with specialized and local supports at educational institutions (e.g., joint meetings with school counsellors). There was also a shift to focus on skills for participating in remote job interviews and learning strategies for remote schooling.

**Table 1c:** Brief report of 5 Virtual NAVIGATE modifications pertaining to the Supported Employment and Education (SEE) role based on FRAME elements (11). Adapted from: Marshall et al. 2021 (34).

FRAME Elements	Brief report from virtual NAVIGATE SEE modifications
**Process**	
**When did the modification occur?**	Similar to IRT, the SEE modifications resulted from the transition to virtual care due to the COVID-19 pandemic, see above.
**Were adaptations planned?**	All modifications were unplanned and reactive, resulting from the sudden onset of the pandemic. For instance, SEE workers could no longer introduce themselves during in-person appointments with the IRT clinician, which instead transitioned to IRT clinicians offering SEE support during IRT sessions and following up with SEE clinicians if the client was interested.
**Who participated in the decision to modify?**	The SCEIS treatment/intervention team made most of the decisions on a clinic/unit level. The SCEIS program leader/clinical manager and individual SCEIS practitioners also participated in the decision to add additional appointments to get to know clients and establish a therapeutic relationship.
**Adaptations**	
**What was modified?**	The context and process of providing supportive employment and education was modified. This included conducting fewer outreach community visits and using phone calls as a reminder when clients did not show for an appointment. These phone calls typically resulted in phone appointments.
**At what level of delivery were modifications made?**	All of the modifications to the SEE role were made within the clinic/unit level at SCEIS.
**What was the type or nature of context or content-level modifications?**	Contextual process changes reflected less outreach community visits compared to in-person care resulting from provincial mandates for lockdown and closures.
**What was the relationship to fidelity?**	Most modifications were fidelity consistent. SEE clinicians were not able to do community visits due to COVID-19 restrictions, which is inconsistent with fidelity.
**Rationale**	
**a. What was the goal?** **b. What were the reasons?**	a. Modifications to the SEE role aimed to increase/maintain client engagement and retention as well as to improve feasibility. b. Reasons largely centred around the outer setting context, namely, the pandemic. Provider reasons for using additional appointments centred on clinical judgement. There were no specific organizational/setting or recipient reasons for this transition.
**Outcome**	
**a. What were the positive outcomes?** **b. What were the negative outcomes?**	a. Meeting virtually allowed for more opportunities to conduct joint appointments (i.e., related to school, employment, counselling) which reduced barriers/increased access for clients to attend SEE sessions (e.g., less travel time). Continuity of care was maintained, especially via phone appointments which were sometimes particularly convenient, and an increase in appointment attendance was observed. b. SEE workers were less able to facilitate connections with employers and counsellors as well as conduct in-person outreach visits or casually drop in, requiring more planning and effort from the client (which may be problematic for job development). There were less opportunities for competitive jobs during pandemic, resulting in more work identifying which jobs were not currently experiencing a hiring freeze.

#### 5.1.5. Modifications for family education and support

As with the IRT component, the majority of modifications affecting provision of family education and support (FEP) were mostly unplanned (Table 1d). Modification decisions were mostly made by the clinical team. Planned adaptations included the creation of additional material (e.g., PowerPoint presentation) to support virtual delivery of psychoeducation groups. As with SEE, the process by which FEP clinicians connected with families was adjusted. Advantages of virtual FEP delivery included increased access to care meetings for caregivers and family members. Some family members and caregivers experienced barriers to using the digital platform and/or internet. Also challenging was how best to facilitate effective communication in a virtual group meeting. To address this, FEP clinicians developed and shared a structure for group meetings with all attendees and offered individual appointments as needed.

**Table 1d:** Brief report of 4 Virtual NAVIGATE modifications pertaining to the Family Education role based on FRAME elements (11). Adapted from: Marshall et al. 2021 (34).

FRAME Elements	Brief report from virtual NAVIGATE FEP modifications
**Process**	
**When did the modification occur?**	Similar to IRT, the FEP modifications resulted from the transition to virtual care due to the COVID-19 pandemic, see above.
**Were adaptations planned?**	The majority of modifications were unplanned and reactive. For instance, family clinicians were no longer able to join the initial or other in-person appointments to introduce themselves, and similar to the SEE role, had to instead connect with the IRT clinician to determine if they have the client's consent to connect with their family members. Planned/ reactive iterative adaptations reflected additional material developed to support virtual psychoeducation groups (i.e., creating a PowerPoint presentation to share on-screen synchronously, and then sent to family members at the end of the meeting).
**Who participated in the decision to modify?**	The SCEIS treatment/intervention team along with the SCEIS program leader/clinical manager made most of the decisions on a clinic/unit level. Individual SCEIS practitioners also participated in the decision to create material to support care being delivered virtually.
**Adaptations**	
**What was modified?**	The context and process of providing family support was modified. This included delivering more NAVIGATE content via phone and offering more videoconferencing groups compared to in-person groups, resulting in more loved ones attending virtually compared to in-person. Structure was also added to virtual groups to facilitate organized communication (using the chat function and “raise hand” function to structure comments and questions).
**At what level of delivery were modifications made?**	All of the modifications to the family clinician role were made within the clinic/unit level at SCEIS.
**What was the type or nature of context or content-level modifications?**	Contextual format changes reflected added virtual groups and the development of virtual material. Contextual process modifications included how the family clinician would connect with the client and their family members during initial and subsequent visits compared to in-person care.
**What was the relationship to fidelity?**	Most modifications were fidelity consistent.
**Rationale**	
**a. What was the goal?** **b. What were the reasons?**	a. Modifications to the family clinician role aimed to increase/maintain client engagement and retention. b. Reasons largely centred on the outer setting context, namely, the pandemic. There were no specific organizational/setting, provider or recipient reasons for this transition.
**Outcome**	
**a. What were the positive outcomes** **b. What were the negative outcomes?**	a. Meeting virtually allowed for a reduction of barriers (time, commuting) and flexibility in attending psychoeducation groups and facilitated balancing other commitments such as working remotely for family members. This led to an increase in group attendance. Phone appointments were particularly convenient for one-on-one sessions. b. For family clinicians, it is harder to connect with all family members virtually in a group session compared to in-person. Other group session challenges included communication procedures (i.e., asking questions, time allotted for each person to speak, managing interruptions, etc.). Some older family members experienced a technology learning curve which was a barrier at the time. Family members also expressed reduced abilities to speak candidly virtually, especially when their loved one (the client) was at home.

#### 5.1.6. Modifications for prescriber

Prescribers were unable to conduct certain activities in a virtual setting as compared to in-person care (Table 1e). This included physical assessments which were postponed early in the pandemic, e.g., monitoring of weight and blood pressure, and assessment of antipsychotic-related movement side effects. To mitigate these challenges and maintain adherence to clinical guidelines, prescribers leveraged community-based resources more often (using local laboratories for bloodwork and community nursing clinics for medication injections).

**Table 1e:** Brief report of 9 Virtual NAVIGATE modifications pertaining to the Prescriber role based on FRAME elements (11). Adapted from: Marshall et al. 2021 (34).

FRAME Elements	Brief report from virtual NAVIGATE Prescriber modifications
**Process**	
**When did the modification occur?**	Similar to IRT, the prescriber modifications resulted from the transition to virtual care due to the COVID-19 pandemic, see above.
**Were adaptations planned?**	The majority of modifications were unplanned and reactive. For instance, more time was needed for administrative work (e.g., faxing/calling-in prescriptions and ordering bloodwork), which limited time spent with the client and typically resulted in additional appointments. Planned/reactive iterative adaptations reflected updates to Mental Health Act (MHA) assessment procedures (i.e., the process of filling out and sending original documentation) and not making significant medication changes (in particular, clozapine) to avoid admissions and intensive follow-up during the first year of the pandemic before vaccines were available.
**Who participated in the decision to modify?**	CAMH leadership made most of the decisions on an organizational level, including to where clients could do their bloodwork, which changed from on-site at CAMH prior to the pandemic, to clients’ local labs post-March 2020. This often resulted in delayed and decreased compliancy to standardized bloodwork follow-up. Prescribers were also no longer able to conduct a fulsome physical assessment virtually. Individual SCEIS practitioners and the treatment team also participated in the decision to use additional appointments to get to know clients and build rapport virtually and to leverage community resources more often to administer injections.
**Adaptations**	
**What was modified?**	The context and process of the prescriber role was modified. This included increasing the frequency of appointments initially during the start of the pandemic and using additional appointments to develop fulsome impressions. Clients were no longer able to complete bloodwork at CAMH at the time of their appointment, in-person self-report questionnaires and physical assessment of side-effects were conducted less frequently.
**At what level of delivery were modifications made?**	Modifications to the prescriber role were made primarily across the CAMH organization as a whole. Some modifications also were made at the clinic/unit level and the individual practitioner level at SCEIS.
**What was the type or nature of context or content-level modifications?**	The contextual process was modified for conducting fulsome physical assessments including bloodwork on-site, assessment of side-effects, and administering self-report questionnaires, which all could no longer continue as a result of the onset of the pandemic. The process for conducting MHA assessments was also modified to a virtual context. Format changes pertained to how appointments were conducted (i.e., videoconference or phone rather than in-person), the use of additional appointments, and allotting added time for increased administrative work.
**What was the relationship to fidelity?**	Roughly half of the prescriber modifications were fidelity consistent. Core elements of the prescriber role that were impacted included fulsome physical assessments, medication changes, and conducting bloodwork on-site at CAMH, which are modifications that are inconsistent with fidelity.
**Rationale**	
**a. What was the goal?** **b. What were the reasons?**	a. Modifications made to the prescriber role aimed to improve feasibility as well as increase/maintain client engagement and retention. b. Reasons largely centred on the outer setting context, namely, the pandemic. There were no specific organizational/setting, provider or recipient reasons for this transition.
**Outcome**	
**a. What were the positive outcomes** **b. What were the negative outcomes?**	a. Meeting virtually allowed for continuity of care with reduced barriers to attending appointments virtually (i.e., reduced travel time and associated costs, decreased stigma/trauma). Prescribers could also check on medication adherence when calling in prescriptions to the pharmacy. Clients often received their injections locally (i.e., at home or at a clinic close by to them). b. Virtual appointments impede physical examinations with clients and missing important clinical presentations by not being able to read non-verbal cues as accurately. This often led to difficulties in building rapport. Challenges with client attention and boundaries arose virtually (i.e., clients engaging in distracting or less appropriate behaviour such as attending appointments while driving or intoxicated, and having others in the household who may be able to listen). There was an increase in last-minute reschedule requests and no-shows during the pandemic as well as adding additional appointments, often resulting in more time spent connecting with clients. Some clients also experienced connectivity issues.

#### 5.1.7. Modifications for the caseload size, team leadership, and training and practice feedback components

Few changes were noted for the three NAVIGATE core components that are not directly related to a clinician role (Table 1f). The team leadership role continued without significant changes, though all meetings were held virtually including supervision and training. Targeted community education decreased, likely related to fewer opportunities for community education as many community events were cancelled/postponed due to the COVID-19 restrictions and educational institutions were busy with the COVID-19 related practicalities including the shift to remote learning with less opportunities for psychoeducation. Training and practice feedback required several changes to the content of the training and practice feedback, e.g., training on virtual care was provided, and practice feedback focussed more on the shift to virtual care delivery and the challenges related to this new method of care delivery. Caseload size did not change, though workload increased significantly for the clinicians and clinical manager due to the added complexity introduced by technology, more frequent appointments, and, anecdotally, improved appointment attendance facilitated by virtual care.

**Table 1f:** Brief report of Virtual NAVIGATE modifications pertaining to the Caseload Size, Team Leadership, and Training and Practice Feedback components based on FRAME elements (11). Adapted from: Marshall et al. 2021 (34).

FRAME Elements	Brief report from e-NAVIGATE Caseload Size, Team Leadership, and Training and Practice Feedback components modifications
**Process**	
**When did the modification occur?**	In general, little modifications occurred to these core components. Similar to the clinician roles, modifications to the Team Leadership, and Training and Practice Feedback resulted from the transition to virtual care due to the COVID-19 pandemic, see above.
**Were adaptations planned?**	The majority of modifications were planned and reactive, such as the additional trainings, e.g., clinician training for improving building engagement with clients in a virtual setting or crisis management.
**Who participated in the decision to modify?**	Most decisions were made with the clinical manager and clinicians.
**Adaptations**	
**What was modified?**	Caseload was not modified and continued to be high. Despite little increase in caseload, workload increased. The leadership role was not modified. Training and Practice Feedback noted increase in training early during the pandemic.
**At what level of delivery were modifications made?**	Most modifications were made at the clinic/unit level and the individual practitioner level at SCEIS.
**What was the type or nature of context or content-level modifications?**	There were no changes to the content of the program.
**What was the relationship to fidelity?**	Mostly fidelity consistent. Core elements that were impacted included fulsome physical assessments, medication changes, and conducting bloodwork on-site at CAMH, which are modifications that are inconsistent with fidelity.
**Rationale**	
**a. What was the goal?** **b. What were the reasons?**	a. Modifications made to the prescriber role aimed to improve feasibility as well as increase/maintain client engagement and retention. b. Reasons largely centred on the outer setting context, namely, the pandemic. There were no specific organizational/setting, provider or recipient reasons for this transition.
**Outcome**	
**a. What were the positive outcomes** **b. What were the negative outcomes?**	a. Meeting virtually allowed for continuity of meetings with reduced barriers to attending appointments virtually (i.e., reduced travel time) b. Virtual appointments impede physical examinations with clients and missing important clinical presentations by not being able to read non-verbal cues as accurately. This often led to difficulties in building rapport. Challenges with client attention and boundaries arose virtually (i.e., clients engaging in distracting or less appropriate behaviour such as attending appointments while driving or intoxicated, and having others in the household who may be able to listen). There was an increase in last-minute reschedule requests and no-shows during the pandemic as well as adding additional appointments, often resulting in more time spent connecting with clients. Some clients also experienced connectivity issues.

### 5.2. Objective 2: fidelity

#### 5.2.1. Fidelity to EPI standards

Table 2 reports item, domain and total fidelity ratings based on the FEPS-FS for two time periods: during 2019, prior to the onset of COVID restrictions and the switch to virtual care delivery, and during 2021, after modifications had been implemented. Of the 33 items in the scale, 4 could not be rated due to lack of data and/or relevance to the Ontario context. For the remaining 29 items, the total mean rating exceeded 4.00 for both time periods, although there were some item level rating differences.

**Table 2:** FEPS-FS assessment results^a^.

Domain	Item	In-person NAVIGATE (Pre- COVID)	Virtual NAVIGATE (during COVID-19 pandemic)
Structure			
2	Participant/provider ratio	5.00	5.00
3	Multidisciplinary team	5.00	5.00
4	Assignment of case manager	5.00	5.00
5	Psychiatrist caseload	5.00	5.00
6	Psychiatrist role on team	5.00	5.00
7	Weekly multi-disciplinary team meetings	5.00	5.00
8	Explicit diagnostic admission criteria	5.00	5.00
10	Duration of FEP program	4.00	4.00
1	Practicing team leader	3.00	3.00
	**Mean domain score**	**4**.**67**	**4**.**67**
Access and continuity (engagement and retention)			
31	Communication between SCEIS and inpatient services	5.00	5.00
32	Timely Contact After Discharge from Hospital	5.00	5.00
12	Early Intervention (Inpatient care prior to admission)	3.00	1.00
13	Timely contact with referred individual	3.00	5.00
11	Targeted community education	2.00	1.00
28	Active engagement (community visits)	1.00	1.00
	**Mean domain score**	**3**.**17**	**3**.**00**
Assessments			
14	Family involvement in initial assessment	4.00	4.00
15	Comprehensive clinical assessment (initial)	5.00	5.00
16	Comprehensive psychosocial assessment (initial)	5.00	3.00
17	Treatment / Care Plan after initial assessment	4.00	5.00
25	Annual formal comprehensive assessment	5.00	5.00
	**Mean domain score**	**4**.**60**	**4**.**40**
Medical			
18	Antipsychotic medication prescription	5.00	5.00
19	Antipsychotic dosing within recommendations	5.00	5.00
24	Supporting Health Management	5.00	5.00
	**Mean domain score**	**5**.**00**	**5**.**00**
Psychosocial Treatment			
21	Client psychoeducation	5.00	4.00
23	Cognitive behavior therapy (CBT)	5.00	5.00
26	Services for Substance Use Disorders	5.00	5.00
27b	Supported education	5.00	5.00
30	Crisis intervention services	5.00	5.00
27a	Supported Employment	3.00	3.00
	**Mean domain score**	**4**.**67**	**4**.**50**
	**Mean overall score**	**4**.**38**	**4**.**28**

^a^ 4 items were not rated (population served, use of clozapine, client retention, family support).

The *program structure domain* mean score did not change between the traditional in-person and virtual NAVIGATE care delivery and it remained high, at 4.67. The *access and continuity domain* mean score declined slightly from 3.17 to 3.00. Within this domain, the *early intervention* item rating decreased from 3.00 to 1.00, indicating an increase in the percentage of clients that were hospitalized prior to entry in the EPI program. The *targeted community education* item rating also decreased from 2.00 to 1.00, indicating fewer community education sessions were being conducted. The rating for *timely contact with referred individual* increased with virtual delivery of NAVIGATE care, indicating more clients were seen within 2 weeks of referral. The *assessment domain* mean score remained high, declining slightly from 4.60 to 4.40 due to lower rating for the initial comprehensive psychosocial assessment item with virtual delivery of NAVIGATE i.e., fewer clients had all components of the comprehensive assessment documented in their consultation note. The *medical treatment domain* mean and item scores did not change over time and remained high, at 5.00. The *psychosocial treatment domain* mean score declined slightly but remained high, at 4.50.

### 5.3. Objective 3: facilitators and barriers

Factors (italicized) affecting virtual EPI delivery are described in Table 3 including their strength and valence. Note that factors were overwhelmingly facilitative, with 10 (27%) showing as mixed. No factors emerged as barriers to re-implementation in this setting and context. Table 3 provides ratings and summaries for each factor.

**Table 3:** CFIR Results.

CFIR Domain/Construct	Rating	Summary Statement
**Intervention Characteristics**		
Intervention Source	+1	Clinicians understood that NAVIGATE was developed in the U.S. and that it is intended to provide evidence-based EPI care that is more formalized, standardized and consistent. Some clinicians stated that according to research, standardized care improves outcomes. NAVIGATE was seen as being implemented due to the desire for more organized and coordinated EPI care.
Evidence Strength and Quality	Mixed	Clinicians felt that NAVIGATE is effective for clients, largely based on their experiences and observations from other clinicians and clients as well as their overall understanding of NAVIGATE. A few mentioned their knowledge about the research behind NAVIGATE. Regarding their initial perceptions of whether NAVIGATE would work virtually, most clinicians admitted that they were doubtful that it would be as effective as in-person. However, over time, they found that it worked equally as well, with some exceptions such as monitoring side effects which requires face to face interaction.
Relative Advantage	+1	Clinicians saw NAVIGATE as augmenting EPI services to a better alternative to how services were previously delivered. With NAVIGATE there is consistency, standardization, and the entire team is involved in client and family care (previously team was disjointed). The virtual delivery of NAVIGATE provided advantages in several ways including: accessibility (clients able to meet more often), flexibility (particularly around school and work), and cost savings (e.g., transportation). Some disadvantages that clinicians identified included not having a platform for clients to complete scales before meeting with the psychiatrist, inequity issues for clients who did not have access to technology and challenges for clinicians in reading body language for assessment purposes.
Adaptability	+2	Adaptations to ensure that the virtual delivery of NAVIGATE worked included implementing and learning how to use WebEx, providing staff with laptops and phones, and converting the manual into PDF fillable forms. Clinicians felt these adaptations were very effective and “working great”. An issue that has not been resolved is the transfer of clients’ self-rated side-effects that they completed on an iPad while waiting to see the psychiatrist.
Trialability	0	Clinicians acknowledged that there was no opportunity to try out the adaptations because there was no time. There was no indication of this being problematic or advantageous.
Complexity	Mixed	For some clinicians, implementing virtual NAVIGATE was regarded as complex, particularly at the beginning because it had to be done quickly with many details to be worked out (e.g., ensuring confidentiality, privacy) and technology was challenging for some people (e.g., family members). However, for others, it was not “terribly difficult” or much extra work because they could rely on others “to figure it out”.
Design Quality and Packaging	+1	Although a few clinicians felt that the materials and supports were not enough at the beginning (e.g., virtual version of the manual, tip sheets) or too much (lots of documents to read and videos to watch), most clinicians felt that they received helpful guidance, information and support from IT as well as from reflective practice meetings.
Cost	Missing	Clinicians could not comment because they were not aware of the costs involved.
**Outer Setting**		
Client Needs and Resources	Mixed	Clinicians’ perceptions of the extent that NAVIGATE meets the needs of clients were mixed. Most clinicians perceived NAVIGATE as being valuable to clients and families based on positive feedback they received, particularly the team approach to care. However, they also noted that for some clients the material was daunting and long, whereas others appreciated the structured approach to their care. Clients with co-morbidities, cultural and language differences and issues accessing the technology were also perceived as barriers to participating in NAVIGATE. To clinicians’ knowledge clients were not consulted on prior to the re-implementation of virtual NAVIGATE.
Peer Pressure	0	Clinicians were not aware of any other sites implementing NAVIGATE prior to SCEIS.
Cosmopolitanism	+1	Clinicians spoke of networking and collaborating with other EPI clinics via ECHO sessions, which informed their NAVIGATE practice. Affiliations with EPION and connections with other mental health agencies and former places of work also influenced clinicians’ NAVIGATE work.
External Policies and Incentives	+2	Provincial best practices and standards for EPI was seen as a major incentive for the implementation of NAVIGATE.
**Process**		
Planning	Mixed	General consensus among clinicians was that there was a lack of planning in the move to virtual delivery, which they recognized as unavoidable due to the sudden need to pivot (i.e., pandemic). Hence at the start, the pivot to virtual delivery was overwhelming. However, clinicians felt that the implementation leaders were the appropriate people and that they did their best to make it as easy and smooth as possible. One participant felt that the SEE role did not receive a lot of guidance. At the time of the interview, most clinicians felt that virtual NAVIGATE was fully implemented.
**Engaging**		
Opinion Leaders	+1	Clinicians felt that the key people who were instrumental in pivoting to the virtual delivery of virtual NAVIGATE worked hard and were collaborative in their approach. They noted several strategies leaders used to encourage and inform staff to move to virtual that entailed numerous emails, links to training, meetings and providing opportunities to ask questions as well as encouraging flexibility in the delivery of NAVIGATE. Clients were informed about the changes through email discussions. Clinicians noted that there was no choice but to move to virtual but made concessions for in person appointments when it was possible.
Formally Appointed Implementation Leaders	+2	Although there was not a lot of planning, the leadership was viewed as collaborative and helpful.
Champions	+1	Identified champions included the implementation leader as well as team members and younger staff who helped others who were not as technically advanced. There was little resistance because everyone knew that it was necessary to pivot to virtual delivery.
External Change Agents	0	Most clinicians could not identify people outside of SCEIS that helped with pivoting to virtual delivery other than the IT department.
Executing	Mixed	Clinicians held mixed opinions about the collaborative execution of the implementation. They spoke of the changes as being a “tsunami”. Some felt that their perspective was sought via team “huddles” and problem-solving discussions as well as opportunities to pose questions to the implementation leaders. Others felt that they were “told” about the programs and systems to use and thus it was more instructive than collaborative.
Reflecting and Evaluating	Mixed	Some clinicians spoke of receiving feedback about what was working and what was not working, as well as statistics about engagement (no shows, who they were seeing) that included discussions and reflections on the information. Others received informal feedback (i.e., no statistics) and others did not recall receiving any specific feedback about virtual delivery.
**Inner Setting**		
Structural Characteristics	Mixed	CAMH was seen as a large, resource-intense setting and hence staff were provided with laptops, phones and rooms for private meetings with clients (virtually as well as in-person) that positively impacted the move to virtual delivery. Areas that still needed changes included finding a way for clients to input their information (without compromising confidentiality) to use the modules effectively as well as improvements to the charting system (electronic).
Networks and Communications	+2	Clinicians mentioned that there were multiple and continuous channels of communication via emails, online team meetings, sharing links to resources, updated policies and problem-solving including communication outside of SCEIS with other EPI sites via ECHO. Although the volume of new information and communications was perceived as overwhelming, it was generally recognized that it was necessary in order to support the transition to virtual delivery of NAVIGATE within days.
Culture	+2	Clinicians regarded the culture of SCEIS as highly positive, collaborative, warm, healthy, supportive, client-centered, and acknowledged that it impacted positively the transition to the online delivery of NAVIGATE. Working together as a team and being focused on delivery the highest quality care possible were perceived as key contributors to the success of transition to virtual NAVIGATE.
**Implementation Climate**		
Tension for Change	+2	Clinicians unanimously noted that there was high tension for change for NAVIGATE – in other words, a program like NAVIGATE was highly needed because of it imposed consistency in delivering care, a holistic and standardized approach, multiple roles with clear scope of practice that benefitted various areas of need for clients.
Compatibility	Mixed	The extent to which virtual NAVIGATE fitted with the existing structures and workflows was perceived as mixed; overall, the virtual delivery of NAVIGATE was compatible with the existing flows but certain roles noted limitations such as poor linkage between virtual NAVIGATE and the charting system, the function of conducting and including assessments virtually, doing injections and benefitting from administrative support.
Relative Priority	+2	The transition to virtual delivery of NAVIGATE was unambiguously perceived as the main priority by all clinicians. There were no other competing priorities and all clinicians fully dedicated their time and attention to the virtual delivery of NAVIGATE, which contributed to its success.
Organizational Incentives and Rewards	+1	There were several incentives noted for both clients and clinicians; for clients, these included the convenience of accessing care, which increased participation, reduced costs related to parking, transportation and time, increased flexibility. For clinicians, Covid and the urgent need to find a way to deliver care to clients was noted as the main incentive. Many clinicians also mentioned that their efforts were recognized by their manager.
Goals and Feedback	0	Most clinicians were not aware of any targets set for the virtual delivery of NAVIGATE and this did not appear to influence their performance. Clinicians were aware of the research component of NAVIGATE and participated in focus groups to share their experiences. Some talked about internal team meetings as an opportunity to share feedback on NAVIGATE or its virtual delivery.
Learning Climate	+1	Overall, clinicians perceived SCEIS’ learning climate positively and acknowledged that it was encouraging of learning and taking on new initiatives. Clinicians valued the availability of multiple learning opportunities, both internally and externally and the support for participation in these opportunities.
**Readiness for Implementation**		
Leadership Engagement	+2	Clinicians unanimously believed that there was support from leadership for the virtual delivery of NAVIGATE and multiple discussions regarding what was needed, special considerations for virtual delivery of care (e.g., privacy; when in-person was essential, role-specific tasks such as who monitors side effects) and that leadership was on board and engaged in the transition process.
Available Resources	+1	Clinicians recognized the availability of many sources of information and supports (e.g., WebEx support, tele-mental health, PSSP, educational services, internal team, etc.) and overall having the resources needed to perform their role successfully. Some clinicians did not receive the original NAVIGATE training and perceived this as a limitation. They valued getting laptops early in the process, which was essential to the virtual transition, but mentioned that access to cell phones was delayed.
Access to Knowledge and Information	Mixed	With respect to access to knowledge and information related to e-NAVIGATE, clinicians described mixed feelings and experiences: there was no formal training, time did not allow for this, but there were multiple resources available to support the transition via links, training videos and emails. The amount of information to be accessed, absorbed and implemented in a very short period of time made the initial experience overwhelming for many clinicians. This improved with time.
**Characteristics of Individual Clinicians**		
Knowledge and Beliefs about the Intervention	+1	Clinicians regarded the NAVIGATE model positively and valued the evidence base and the holistic approach. With respect to its virtual delivery, clinicians believed that it had great advantages and met the needs of a large number of clients but it could not be the only way to deliver care. For instance, some roles (e.g., psychiatrists) noted the need to have in-person assessments periodically to have a more accurate sense of the clients’ status.
Self-Efficacy	+1	Overall, clinicians reported a sense of self-efficacy in delivering NAVIGATE virtually. For many, this confidence stemmed from feeling effective in the delivery of NAVIGATE in person, which provided a solid basis for the transition to the virtual delivery.
Individual Stage of Change	+1	Clinicians talked about feeling prepared to deliver NAVIGATE virtually but feeling slightly hesitant and overwhelmed at the start given the abrupt transition. With time, there was an increased sense of preparedness with practice and continuous refinement of the online resources to support staff.
Individual Identification with the Organization	+2	There was a general consensus among clinicians that their commitment to SCEIS strongly and positively influenced their interest in learning, taking on new initiatives, adapting to change, and providing the best care for clients. It was noted that the transition to the virtual delivery of NAVIGATE ultimately was an exercise in change management and was closely tied to how the employer was perceived.
Other Personal Attributes	Mixed	Clinicians discussed mixed thoughts and experiences related to the transition to virtual delivery of NAVIGATE and alignment with their preferred learning style. Some appreciated the convenience of accessing materials online and learning at their own pace; in contrast, others found it distracting and ineffective to be trained online. Overall, clinicians reported high levels of motivation to make virtual delivery of NAVIGATE work.
**Characteristics of Clients**		
Beliefs and Experience	+1	Based on the feedback received and their own observations, clinicians believed that the virtual NAVIGATE experience was positive for both clients and their families. Overall, the virtual delivery of NAVIGATE brought great advantages stemming from the convenience of accessing care. Clinicians believed that virtual NAVIGATE facilitated fewer no-shows and increased access to care and client engagement. A period of adjustment was needed at the start of the transition as clients and their families, similar to the healthcare providers, had to learn the details of the online system.
**Success**		
Success	+2	The transition to the virtual delivery of NAVIGATE was perceived as successful, with the team being able to adapt smoothly to the new demands of virtual delivery of NAVIGATE and to learn and work together as a team. Clinicians unanimously recommended continuing the virtual delivery of NAVIGATE while recognizing that in an ideal scenario the clients would have a choice for in-person or virtual NAVIGATE, to fit their needs. Having a virtual delivery option was perceived as a way to increase access to care across the country.

CFIR, consolidated framework for implementation research; ECHO, extension for community outcomes; EPION, early psychosis intervention ontario network; PSSP, provincial system support program.

#### 5.3.1. Intervention characteristics

*Adaptability* (+2) of NAVIGATE to the virtual context was most strongly associated with its re-implementation (see Table 3). Adaptations to ensure that the virtual delivery of NAVIGATE was appropriate and effective included implementing and learning how to use the Cisco Webex platform, providing clinicians with laptops and phones, and converting the intervention manual into PDF fillable forms. Clinicians felt these modifications were very effective and “working great”. One issue that remained unresolved was the transfer of client-rated side-effects completed on an iPad while waiting to see the psychiatrist.

NAVIGATE was originally implemented due to the desire for more organized and coordinated EPI care throughout Ontario (*Intervention Source* +1). Virtual delivery of NAVIGATE provided advantages in several ways including accessibility (clients able to meet more often), flexibility (scheduling around school and work), and cost savings (e.g., no need for transportation). Some disadvantages included not having a platform for clients to complete a questionnaire before meeting with the psychiatrist, inequity issues for clients who did not have access to virtual care, and challenges for clinicians in reading body language for assessment purposes (*Relative Advantage* +1).

Although a few clinicians felt the materials and supports were either not supportive enough at the beginning of the re-implementation (e.g., fillable PDF version of the manual, tip sheets) or provided too much information to absorb (lots of documents to read and videos to watch), most felt that they received helpful guidance, information and support from IT personnel as well as from the reflective practice meetings (*Design Quality and Packaging* +1).

Two intervention factors had mixed ratings. Clinicians felt NAVIGATE was effective for clients, largely based on their experiences and observations shared from other clinicians and clients, as well as their overall understanding of intervention (*Evidence Strength and Quality*, mixed). A few clinicians mentioned they were knowledgeable about the research evidence underlying the intervention. Yet, most clinicians initially felt doubtful that NAVIGATE would be as effective virtually as in-person. With time, however, they found that it worked equally well with the exception of monitoring side effects, which required face to face interaction.

With respect to *Complexity* (mixed), some clinicians found re-implementing NAVIGATE for virtual delivery to be difficult, particularly at the beginning, because it had to be done quickly with many details to be worked out (e.g., ensuring confidentiality, privacy). As well, the technology was challenging for some users (e.g., family members). Other clinicians reported that it was “not terribly difficult” or not much extra work to re-implement because they could rely on others “to figure it out”.

#### 5.3.2. Outer setting factors

Provincial best practices and EPI standards were seen as providing a major incentive for the implementation of NAVIGATE (*External Policies and Incentives*, +2). Somewhat less facilitative was the experience of networking and collaborating with other EPI services via EPI-SET ECHO training sessions, which are intended to inform NAVIGATE practice (16). The ECHO (Extension for Community Healthcare Outcomes) model connects geographically dispersed healthcare providers in online communities of practice with the aim of increasing healthcare access (35). Affiliations with EPION and with other mental health agencies and former places of work also influenced clinicians' work (*Cosmopolitanism*, +1).

The extent to which NAVIGATE met *Client Needs* was mixed among respondent clinicians. Most perceived NAVIGATE as valuable to clients and families, based on the positive feedback they received, particularly the structured and team approach to care. However, they also noted that for some clients, the material was daunting and lengthy. Cultural and language differences, clients having comorbidities, and issues accessing the technology were also perceived to be barriers to participating in NAVIGATE. The rapid pivot to virtual delivery also meant there was no time to consult clients about the change. *Peer Pressure* (0) was perceived as neither a barrier nor a facilitator since no other provider organizations were delivering NAVIGATE at that time of this study.

#### 5.3.3. Process factors

The strongest facilitator for re-implementation was the presence of *Formally Appointed Implementation Leaders* (+2). Although there was not a lot of pre-pandemic planning, the leaders were viewed as collaborative and helpful. The presence of *Champions* (+1) and *Opinion Leaders* (+1) was also facilitative. Clinicians felt that key people who were instrumental in pivoting to the virtual delivery of NAVIGATE worked hard and were collaborative in their approach. They noted several strategies leaders used to encourage and inform clinicians to move to virtual delivery of care including numerous emails, links to training, meetings, and providing opportunities to ask questions as well as encouraging flexibility in the delivery of NAVIGATE. Clients were informed about changes through email discussions. Clinicians further noted that there was no choice but to move to virtual care delivery but made concessions for in-person appointments when it was possible.

Clinicians held mixed opinions about the *Executing* of the re-implementation. They spoke of the changes as being a “tsunami”. Some clinicians mentioned they were consulted via team “huddles”, problem-solving discussions and opportunities to pose questions to the implementation leaders. Others felt that they were “told” about the changes and that execution was more instructive than collaborative.

The consensus among clinicians was that there was a lack of *Planning* (mixed) in the move to virtual delivery, which they recognized as unavoidable due to the sudden need to maintain service in the pandemic. Initially, the pivot to virtual delivery was overwhelming. However, clinicians felt that the implementation leaders were the appropriate people to lead the way and that they did their best to make it as easy and smooth as possible. One clinician felt that the SEE role did not receive a lot of guidance. At the time of the interview, most clinicians felt that virtual NAVIGATE had been fully re-implemented.

Opportunities for *Reflecting and Evaluating* were also mixed. Some clinicians spoke of receiving feedback about what was working and what was not working, as well as statistics about engagement (clients who did not attend their appointment, who they were seeing) that included discussions and reflections on the information shared. Others received informal feedback (i.e., no statistics) and others did not recall receiving any specific feedback about how virtual delivery was going.

#### 5.3.4. Inner setting factors

*Structural Characteristics* (mixed) of the organization were noted as having both positive and negative influences on re-implementation. A strength was that CAMH is a large, resource-intensive setting where staff were provided with laptops, mobile phones, and rooms for private meetings with clients (virtually as well as in-person). Barriers were that clients were unable to input personal information when using the virtual modules without compromising confidentiality, and improvements are needed to the electronic health record.

CAMH as a setting was also highly facilitative for re-implementation due to its Culture (+2) and *Networks and Communications* (+2). Clinicians regarded the workplace culture as highly positive, collaborative, warm, healthy, supportive, client-centered, and acknowledged that it impacted positively on the transition to virtual delivery of NAVIGATE. Working together as a team and focusing on delivering the highest quality care possible were perceived as key contributors to the success of the re-implementation. The multiple and continuous channels of communication via emails, virtual team meetings, sharing links to resources, updated policies and problem-solving including communication outside of CAMH with other EPI sites via ECHO were perceived as very supportive. Although the volume of new information and communications was overwhelming, it was generally recognized as necessary to support the transition to virtual delivery within a matter of days.

Within the *Implementation Climate*, specifically *Tension for Change* (+2) and *Relative Priority* (+2) were the strongest facilitators in this domain. Clinicians unanimously noted a high tension for change for NAVIGATE because it provided consistency in delivering care, a holistic and standardized approach, and its multiple roles had a clear scope of practice that benefitted various client needs. The transition to virtual NAVIGATE was unambiguously perceived as the main organizational priority by all clinicians. Competing priorities fell to the wayside and all clinicians fully dedicated their time and attention to the virtual delivery, which contributed to its success.

*Organizational Incentives and Reward* (+1) were also facilitative with several incentives noted for both clients and clinicians. Client incentives included the convenience of accessing care which increased participation, reduced time, parking and transportation costs, and increased flexibility. Clinicians were strongly motivated by the urgent need to find a way to maintain care delivery in the face of pandemic restrictions. Many also mentioned that their efforts to re-implement were recognized by their clinical manager.

The *Learning Climate* (+1) at SCEIS was perceived positively and as encouraging of learning and taking on new initiatives. Clinicians valued the availability of multiple learning opportunities, both internally and externally, and the supports provided for participating in these opportunities.

*Leadership Engagement* (+2) was the strongest readiness facilitator. Clinicians unanimously believed there was support from leadership for the virtual delivery of NAVIGATE. Multiple discussions were held regarding what was needed, special considerations for virtual delivery of care were put in place (e.g., privacy; when in-person was essential, role-specific tasks such as who monitors side effects) and leadership were on board and engaged in the re-implementation process.

Re-implementation was supported by *Available Resources* (+1) including many sources of information and supports to ensure clinicians had the resources needed to perform their role successfully (e.g., Webex support, Virtual Mental Health and Outreach program, educational services, internal team, etc.). Some clinicians had not received the original NAVIGATE training in the initial implementation and perceived this as a limitation. Clinicians valued getting laptops early in the process, which was essential to the virtual transition, but noted that access to cell phones was delayed.

Experience with *Access to Knowledge and Information* was mixed as re-implementation did not include formal training due to the rapidity of the pivot. There were, however, multiple resources available to support the transition via links, training videos and emails. The amount of information to be accessed, absorbed and implemented in a very short period of time made the initial experience overwhelming for many clinicians but this improved with time.

#### 5.3.5. Characteristics of clinicians

The most facilitative factor related to the clinicians was their *Individual Identification with the Organization* (+2). There was a general consensus among clinicians we interviewed that their commitment to CAMH strongly and positively influenced their interest in learning, taking on new initiatives, adapting to change, and providing the best care for clients. It was noted that the transition to the virtual delivery of NAVIGATE ultimately was an exercise in change management and was closely tied to how the employer was perceived.

Clinicians’ *Knowledge and Beliefs about the Intervention* (+1) was also supportive of re-implementation. Clinicians regarded the NAVIGATE model positively and valued the evidence base and the holistic approach. They viewed virtual delivery as advantageous but some clinicians (i.e., psychiatrists) noted the need to have in-person assessments periodically to have a more accurate sense of the clients' status.

Clinicians reported a sense of *Self-Efficacy* (+1) in delivering NAVIGATE virtually. For many, this confidence stemmed from feeling effective in the delivery of NAVIGATE in person, which provided a solid basis for the transition to virtual delivery. They felt prepared to deliver NAVIGATE virtually (*Individual Stage of Change* +1), but also slightly hesitant and overwhelmed at the start given the abrupt transition. With time, there was an increased sense of preparedness with practice and continuous refinement of the online resources to support clinicians. Participants discussed mixed thoughts and experiences related to the transition to virtual delivery of NAVIGATE and alignment with their preferred learning style. Some appreciated the convenience of accessing materials online and learning at their own pace; in contrast, others found it distracting and ineffective to be trained online. Overall, clinicians reported high levels of motivation to make virtual delivery of NAVIGATE work.

#### 5.3.6. Client characteristics

Clinicians believed virtual navigate provided a positive experience for both clients and their families. The virtual delivery of navigate was very advantageous for continuing to access care when in-person care could not be delivered. There were fewer no-shows, increased access to care and better client engagement. A period of adjustment was needed at the start of the transition as clients and their families had to become familiar with the digital platform, as did the clinicians.

The transition to the virtual delivery of NAVIGATE was *Perceived as Successful* (+2), with the team being able to adapt smoothly to the new demands and to learn and work together as a team. Clinicians unanimously recommended continuing with virtual delivery of NAVIGATE while recognizing that in an ideal scenario, clients would have a choice of in-person or virtual delivery to fit their needs and preferences. Having a virtual delivery option was perceived as a way to increase access to care across the country.

### 5.4. Stakeholder engagement

The stakeholders, including youth and family with lived experiences, front-line clinicians, and clinical leads, participated consistently and meaningfully throughout the course of this study.

In the initial phases, all stakeholders participated in the grant application and development of the practice profile with front-line clinicians (22, 36). For objective 1, Modifications, front-line clinicians, clinical leads and youth and family with lived experiences participated in monthly meetings to explore and review modifications that occurred during the shift to virtual care delivery. Following these meetings, trainings were organized in collaboration with clinical staff, leadership and youth and family members with lived experience. Youth with lived experiences also contributed to the development of the web-based resources to enhance engagement. Regarding objective 2, Fidelity, feedback from front-line clinicians and clinical leads informed the fidelity ratings. Regarding objective 3, Implementation Facilitators and Barriers, front-line clinicians and clinical leads participated in the interviews.

Furthermore, youth and family with lived experience, front-line clinicians and clinical leads contributed to team discussions on data interpretation and development of a knowledge translation plan and products.

## 6. Discussion

In this mixed methods study investigating the unplanned shift to virtual delivery of EPI care, we identified several modifications required to deliver the NAVIGATE program virtually by using the NAVIGATE practice profile and the FRAME framework. We discussed the potential impact of these modifications on fidelity and outcomes during structured meetings with clinicians, revised the practice profile, and captured modifications using the FRAME. We then formally evaluated impacts on fidelity to the provincial EPI-standards with a validated assessment tool (FEPS-FS) prior to and after the modifications were made. We investigated implementation facilitators and barriers for the virtual delivery of NAVIGATE with clinicians and identified several contextual factors that were critical to re-implementation of NAVIGATE. To our knowledge, this is the first study to describe a re-implementation process this comprehensively. We summarize overall results and experiences with this re-implementation process, strengths and limitations of the approaches we used, and opportunities and needs for future research.

### 6.1. Modifications

Regarding the first aim of the study, the identification of modifications needed for virtual EPI care, we identified several cross-cutting and role-specific modifications. Most of these modifications were adaptable, though some challenges were identified that could not be mitigated in a virtual setting (e.g., conducting physical assessment).

Our assessment of the modifications needed to support virtual care delivery is largely similar to two recent studies that also describe the shift to virtual care in early psychosis coordinated specialty care programs (37, 38). McCormick and colleagues investigated the pandemic-driven shift to continue care delivery via videoconference and phone at 23 sites across Texas, US (37). Their results show many sites lacked training, resources, policies and procedures to shift to virtual care, and the challenges that were identified included limited capacity to deliver community-based outreach, family engagement, and vocational support, and difficulties with access and connectivity for clients. Similar to the modifications we identified in our study, organizations provided training to support staff early on in the pandemic, leveraged virtual tools e.g., e-mailing clinical worksheets, sharing mobile apps, and sharing other resources such as videos, and reimbursed virtual care - an important facilitator for virtual care delivery (37). Similarly, Meyer-Kalos and colleagues explored challenges and solutions in the shift to virtual care delivery across several EPI services in the United States, Israel, and China (38). These authors also highlighted the importance of implementing procedures to provide care virtually, adapting appointments times and duration, and adapting materials for digital use. They describe specific challenges and mitigating strategies at the clinician-level per NAVIGATE role, such as challenges for SEE clinicians associated with the COVID-19 constricted labor market and unavailability of outreach visits (38, 39). They described modifications similar to ours, such as shifting focus to practicing skills for remote learning and working and conducting job interviews remotely. Regarding the prescriber role, both our study and Meyer-Kalos’ reported challenges with follow-up for medication benefits and side-effects, and a reluctance by prescribers to make changes to medication, particularly switching to clozapine because it requires monitoring with blood tests that were challenging to obtain during the pandemic (38). Mitigating strategies also overlap across our studies, with increased frequency of appointments and involvement of family members to improve monitoring of medication (38).

There are similarities between the modifications and mitigating strategies we identified in similar studies in the child and youth health mental services sector in Ontario (9). Common strategies included provision of software and hardware, clinician training in software to provide virtual care, adapting materials, offering phone sessions and adding text message-based support to address accessibility issues, development of safety protocols, and breaking sessions into smaller segments to increase client engagement. Clinicians were encouraged to engage in self-care activities and some clinics installed flexible hours of service to accommodate clients' and clinicians' other responsibilities (9). The similarities across settings surfaced several cross-cutting modifications needed for delivery of virtual care as well as specific adjustments related to NAVIGATE role-based core components.

Coding of modifications in the FRAME (11) highlighted that modifications were mainly initiated by the COVID-19 pandemic. Modifications occurred at different levels, ranging from the SCEIS team to the CAMH organization to the provincial government. Decisions underlying the modifications were also made at these levels, by individual clinicians, clinic manager, and organizational leadership. Of note is that, the COVID-19 pandemic did not only trigger this pivot to virtual care but the COVID-19 related effects were wide-ranging, from impacts on the health systems organization, e.g., reduced access to primary care, but also impacting clients in the reduced opportunity for finding work or attending school remotely, which is reflected in several modifications.

### 6.2. Fidelity

Fidelity assessment with the FEPS-FS revealed that the majority of EPI items (23/29) were rated as satisfactorily or fully implemented, and that the core structure of the NAVIGATE program was strongly preserved despite modifications for virtual delivery. These positive results may be related to the extra training and support clinicians received to facilitate re-implementation from the onset of the pandemic.

Compared to the fidelity assessment of in-person NAVIGATE care, the level of program delivery was maintained for many of the assessed items and improved in several areas in the virtual context. The results for the domain *access and continuity* were mixed, e.g., item scores on timely contact with the referred individual improved, but more new clients had experienced inpatient psychiatric admissions prior to entering the EPI program, and delivery of targeted community education events decreased. The faster connection to a clinician after referral could reflect improved access to care virtually (fewer missed appointments), reduced clinic waitlist, and greater client flexibility to meet during the daytime (individuals were less constrained by work or school hours). On the other hand, most clients experienced an inpatient admission before their admission to NAVIGATE, and this proportion increased compared to in-person care before the COVID-19 pandemic.

Increased inpatient admission could be related to the COVID-19 pandemic. Worsening mental health symptoms and/or increased substance use during the pandemic (40, 41) could have led to more hospitalizations for psychosis (42) or a decline in visits to the primary care providers who could have otherwise referred for outpatient early intervention care (43). As well, there may have been fewer opportunities for youth to connect with their wider support system, such as teacher or coaches, who might otherwise have detected mental health issues and supported them with finding appropriate supports/early treatments.

Additionally, targeted supports for community-based education and employment also decreased. This was a challenge for the CAMH EPI program before the pandemic because of how hospital-based care is organized. The decrease in educational supports also stemmed from the cancellation or postponement of community education due to COVID-19 restrictions, and educational institutions prioritized COVID-19 related practicalities including the shift to remote learning.

Despite reservations voiced by staff about the virtual delivery of medical care in the FRAME discussions, fidelity ratings for *health management* in the medical care domain remained high. Fidelity feedback for health management suggested that mitigation strategies were identified such as leveraging alternatives to physical assessment (e.g., measure weight at home or blood pressure at pharmacy or primary care practice). Also, while it is possible that physicians were more cautious about medication management, prescribing remained within recommended guidelines which is what the fidelity review assesses.

Implementation remained high for *delivery of psychosocial treatments*, which aligns with efforts to sustain client retention by offering different options for connecting, shifting to shorter, more frequent meetings, and synchronously sharing fillable PDFs. As captured in the FRAME, most modifications were described as fidelity-consistent, which is reflected by the “fully implemented” fidelity scores. To our knowledge, there are no other published studies investigating fidelity for a virtual comprehensive EPI care program compared to in-person care. There are, however, several studies that report on treatment fidelity for virtual delivery compared to in-person delivery of a structured psychosocial intervention in other populations. In these publications, there was no evidence that virtual delivery achieved worse fidelity compared to in-person delivery (44–46).

### 6.3. Facilitators and barriers

CFIR interviews surfaced several factors that facilitated the re-implementation of virtual care. The most salient facilitators were *adaptability* of NAVIGATE, *external policies and incentives*, and the *tension for change* brought on by the COVID-19 pandemic. *Implementation leaders* were also highly facilitative, despite the abrupt shift and limited time for planning. Workplace *culture*, clinicians' *identification with the organization*, and the transition to virtual NAVIGATE becoming a strong *relative priority* in the organization.

Few barriers were mentioned, but clinicians noted that virtual delivery did not always align with *client needs and resources*. Some clients found the intervention related material challenging to get through, while others were challenged by cultural and language differences, co-morbidities, issues accessing technology and challenges with adequately monitoring side-effects in a virtual setting.

These results are largely in line with a recent study exploring the pandemic-related transition to virtual care across child and youth mental services in Ontario (9). Using a multi-level mixed method design and CFIR interviews, Danseco identified several facilitators including staff engagement and motivation, provision of enabling software and hardware, leadership support, and training activities (9). Clinicians also mentioned the positive impact of collaboration and having a champion or community of colleagues for learning virtual care delivery together. Barriers in the Danseco study included internet connection issues, lack of resources, and privacy concerns (9). Clinicians also noted fatigue from engaging in online sessions and a feeling of isolation from their colleagues. The authors concluded that overall, many service providers had similar experiences implementing virtual care. With the appropriate support, infrastructure, and resources, many clinicians and clients found virtual delivery of care acceptable and would like to continue using it or having it as an option (9).

Our findings also align with factors associated with implementation success across a diverse array of settings and interventions, including weight management in a large integrated U.S. healthcare system, an e-health application in Norway, and a Canadian study of a maternal and child health intervention undertaken in Mali and Ethiopia (47).

### 6.4. Re-implementation process

Use of the NAVIGATE practice profile and the FRAME to identify modifications facilitated a structured, explicit and comprehensive assessment of modifications in a dynamic context that could have negatively impacted care delivery (11, 48). Taking stock of modifications to core intervention components is crucial for understanding fidelity and effectiveness outcomes (18). The addition of clinician-reported barriers, mitigating strategies and impacts to our practice profile enabled us to track what strategies were used to reduce potentially negative impacts. This approach tracking and using data “along the way” to inform subsequent adaptations (e.g., updates to training, material) contrasts with more linearly designed studies that conduct fulsome impact assessments prior to refining and evaluating an adapted version of an intervention that is hypothesized to fit better (49). Rapid and iterative assessments of modifications and impacts provided a great advantage to optimizing re-implementation, especially when unplanned modifications could negatively impact outcomes (11, 48). A similar stepwise process of revising/developing policies and workflows, providing training, reflecting/evaluating, and taking steps for further improvement during the abrupt shift to virtual care in the pandemic was also observed in other health care agencies that implemented virtual delivery of care in Ontario (9). Another advantage of using the practice profile was that clarity on the intervention components made it was easy for clinicians to identify where and what modifications were needed and/or had occurred.

A disadvantage of our approach was that some of the FRAME domains overlap with the determinant domains of the CFIR, which is less efficient compared to using one instrument only. Other studies also described an overlap between the FRAME and CFIR and decided to reduce certain items of the FRAME for efficiency (50). Furthermore, several of the components of the Process domain of the FRAME were similar between the modifications and were summarized to lessen redundancy. Additionally, the original FRAME framework does not capture the impact of a modification. We added a category of impact and mitigating strategy to the FRAME constructs because systematic consideration of all potential impacts on a range of implementation and intervention outcomes is critical for further optimization of the intervention (51).

Regarding the fidelity assessment, we measured fidelity to the provincial EPI standards with a validated measure, the FEPS-FS. We intend to measure fidelity to the core components of NAVIGATE by reviewing delivery metrics from randomly selected charts, and report the results of thisin a future paper.

### 6.5. Strengths and limitations

To our knowledge this is the first study of the re-implementation of a comprehensive early psychosis intervention for virtual care delivery. We investigated modifications, fidelity to EPI standards, and determinant factors which are the first 3 objectives of our larger, mixed-methods study. Previous studies have investigated satisfaction, and facilitators and barriers to virtual care delivery based mostly on interviews with health care providers (52), though some included client experiences (53, 54). Our study presents a more rigorous approach to investigating re-implementation of a comprehensive intervention during an abrupt shift to virtual care initiated by the demands of the COVID-19 pandemic. Here, we report on the first objectives of the study. In a later paper we will describe client's and clinician's experiences and measures of engagement later to provide a fulsome description of the impact of virtual care delivery of NAVIGATE.

The success of our re-implementation may be unique to the COVID-19 pandemic context. COVID-19 related restrictions to social contacts likely triggered a strong motivation to continue care while adhering to these restrictions, leading to a quick pivot to virtual care delivery. Other key facilitating factors may also be unique to this context, including the support provided by CAMH and the Virtual Mental Health and Outreach program specifically, the adaptability of the NAVIGATE program, and other availability of resources such as materials and funds (30).

Furthermore, the switch to virtual care delivery may have unintentionally created disparities in the mental health care system for people with limited or no access to technology or to the private space needed to attend virtual appointments (55). Relatedly, social isolation may be an unavoidable outcome of virtual care delivery that will require further examination to address. Ongoing remuneration for virtual service delivery remains uncertain and will undoubtably be an important consideration to monitor moving forward.

### 6.6. Future steps

Virtual EPI care has the potential to complement traditional in-person EPI care and improve access in specific contexts, e.g., in remote geographic areas. Improving access to specialty health care is particularly relevant for individuals living in rural and remote communities as they tend to experience poorer health, greater disability, and higher mortality (56). To facilitate equitable care, it will be important to investigate client experiences with virtual care, and to address related barriers stemming from sociodemographic factors that lead to health disparities (55, 57). Following on the results from this work, more research is needed to assess the efficacy and generalizability of virtual EPI care and patient's preferences towards virtual or hybrid care, beyond the COVID-19 pandemic context.

## 7. Conclusion

In conclusion, re-implementation of NAVIGATE for virtual delivery during the COVID-19 pandemic was rapid, unplanned, and complex. Understanding how re-implementation transpired, involved an exploration of barriers, strategies, and impacts across levels of the organization. This study suggests that a comprehensive EPI program can be re-implemented for virtually delivery while maintaining high EPI standards with the appropriate support, infrastructure, and resources. Virtual delivery of NAVIGATE holds promise for increasing access to effective care for youth with psychosis. Going forward, it will be important to ensure future pivots to virtual delivery for NAVIGATE and other interventions maintain equitable care.

## Data Availability

The original contributions presented in the study are included in the article/Supplementary Material, further inquiries can be directed to the corresponding author.
